# Machine learning and multi-omics clustering to map cellular rewiring and immune evasion in ccRCC

**DOI:** 10.1186/s40246-026-01024-8

**Published:** 2026-09-15

**Authors:** Zhe Wang, YingJian Wang, Jiayi Zhang, Yuechang Zhang, Shaoyang Xv, Long Zhang, Feng Wang, Dao Xin

**Affiliations:** 1 Department of Oncology, The First Affiliated Hospital of Zhengzhou University, Zhengzhou, China; 2 Tianjian Laboratory for Advanced Biomedical Sciences, Zhengzhou, China; 3 State Key Laboratory of Metabolic Dysregulation & the Prevention and Treatment of Esophageal Cancer, Zhengzhou, China; 4 Department of Urology, The First Affiliated Hospital of Zhengzhou University, Zhengzhou, China; 5 Department of Urology, Zhongda Hospital, School of Medicine, Southeast University, Jiangsu 210009 Nanjing, China

**Keywords:** Spatial immune exclusion, Multi-omics signature, Machine learning integration, Drug sensitivity, Tumor microenvironment

## Abstract

Immune checkpoint blockade (ICB) efficacy in clear cell renal cell carcinoma (ccRCC) is limited by tumor microenvironment (TME) heterogeneity. Because traditional bulk-derived models lack spatial resolution, we developed an integrated framework connecting macroscopic survival risks to microscopic TME structures. We applied ten algorithms to establish multi-omics subtypes and evaluated 101 machine-learning combinations across three independent cohorts to generate a Consensus Machine Learning-driven Signature (CMLS). The signature’s spatial and cellular origins were decoded using spatial transcriptomics (ST) and a 140,000-cell scRNA-seq atlas. Expression of key genes was experimentally validated via RT-qPCR in 17 paired ccRCC clinical tissues. We identified two molecular subtypes with distinct clinical and epigenetic profiles. SuperPC optimization yielded a 24-gene CMLS serving as an independent prognostic factor. scRNA-seq and ST deconvolution revealed these signals predominantly originate from cancer-associated fibroblasts (CAFs) and malignant epithelial cells, which collaborate to drive spatial immune exclusion. RT-qPCR confirmed significant overexpression of five core CMLS genes in ccRCC versus adjacent normal tissues. Low CMLS scores correlated with enhanced ICB responsiveness, whereas high-CMLS tumors demonstrated specific vulnerability to dasatinib and dabrafenib. The CMLS translates spatial immune-exclusion dynamics into a quantifiable metric, outperforming tumor mutational burden in predicting ICB benefits, providing a robust tool for patient stratification in ccRCC.

**Supplementary Information:**

The online version contains supplementary material available at https://doi.org/10.1186/s40246-026-01024-8.

## Introduction

Clear cell renal cell carcinoma (ccRCC) is defined by profound molecular heterogeneity and remains the most lethal urological malignancy [1, 2]. Although immune checkpoint blockade (ICB) has revolutionized its clinical management, therapeutic responses remain highly unpredictable [3, 4]. The complex, actively regulated tumor microenvironment (TME) of ccRCC fundamentally governs this immune evasion [5, 6]. Traditional genomic metrics, such as tumor mutational burden (TMB), are insufficient for stratifying ccRCC patients [7], as they fail to capture the dynamic intercellular crosstalk within the TME [8].

Although numerous prognostic models exist for ccRCC [9, 10], their clinical reliability is severely restricted by methodology. The majority of these tools rely exclusively on single-omics data and solitary algorithms. This one-dimensional profiling completely misses the VHL-driven epigenetic and metabolic shifts at the core of ccRCC pathogenesis [11, 12], inevitably causing model overfitting and poor generalization. Beyond these algorithmic blind spots, conventional bulk-sequencing signatures treat the entire tumor as a uniform mixture. Such homogenization erases essential single-cell and spatial resolution [13, 14]. Without this microscopic context, researchers cannot determine which exact cell states encode the macroscopic risk signals, nor can they observe how these cells physically coordinate to drive immune evasion. Ultimately, this lack of biological grounding prevents the translation of abstract mathematical scores into actionable drug targets [15].

To overcome these systemic limitations, we established an integrative multi-scale framework combining extensive computational modeling with targeted experimental validation. Initially, ten diverse clustering algorithms were applied to multi-omics data to identify stable molecular subtypes. Rather than depending on a single methodology, we systematically evaluated 101 machine-learning combinations to construct the Consensus Machine Learning-driven Signature (CMLS). By projecting this bulk-derived metric onto high-resolution single-cell and spatial transcriptomic atlases, we precisely localized the cellular sources of the CMLS and mapped their topological rewiring within the TME. Crucially, to validate our in silico findings, we performed quantitative real-time PCR (RT-qPCR) on an independent clinical cohort of 17 paired ccRCC tissues, experimentally confirming the pronounced expression dysregulation of core CMLS hub genes. Validated across multiple cohorts, the CMLS demonstrates superior accuracy over standard biomarkers in predicting ICB efficacy and highlights distinct pharmacogenomic vulnerabilities, providing a robust, experimentally supported blueprint for precision oncology in ccRCC.

## Methods

### Data acquisition

The GEO repository provided single-cell RNA sequencing (scRNA-seq) profiles under accession GSE207493. Bulk transcriptomic datasets originated from multiple public platforms, including the TCGA-KIRC project via the UCSC Xena browser, the CPTAC-ccRCC database, the IMvigor210 clinical trial, and two ArrayExpress cohorts (E-MTAB-1980, E-MTAB-3218). Spatial transcriptomics (ST) records (record ID: 8063124) were obtained directly from the Zenodo repository to complete the multi-scale dataset.

### Multi-omics data integration and multicenter cohort preprocessing

The TCGA-KIRC dataset served as the foundational multi-omics cohort, providing paired RNA expression, DNA methylation, somatic mutations, and clinical annotations. Four external independent cohorts (CPTAC-ccRCC, IMvigor210, E-MTAB-1980, and E-MTAB-3218) expanded this analytical pool. Raw RNA-seq expression matrices underwent conversion to transcripts per million (TPM). Microarray data processing relied on the limma R package for background noise correction, log2 transformation, and quantile normalization. Duplicated probes were collapsed by retaining the maximum expression value per gene locus. Boxplot visualizations confirmed the initial sample distribution consistency. The ComBat function from the sva package then neutralized technical batch effects across different sequencing platforms, and principal component analysis (PCA) confirmed the clean integration of these multi-center datasets.

### Consensus clustering of multi-omics profiles

Aligning cases by shared sample identifiers generated a core multi-omics cohort of 261 patients. Their corresponding TPM matrices received a log2 transformation. Methylation array processing isolated probes specifically targeting promoter CpG islands. Mutation screening retained only functionally relevant non-synonymous genomic alterations, encompassing missense/nonsense mutations, indels, and splice-site modifications.

The MOVICS package handled the feature selection phase. The getElites function calculated the median absolute deviation ("mad" method) to capture the top 1000 highly variable continuous features across mRNAs, lncRNAs, miRNAs, and methylation profiles. Univariate Cox regression ("cox" method) filtered this pool, isolating features significantly linked to patient prognosis (P < 0.05). Discrete mutation profiling focused on the top 5% most frequently altered genes using the "frequency" parameter.

Subgroup stability evaluation relied on the getClustNum function, which outputted the Silhouette score, Gap statistic, and Clustering Prediction Index (CPI). These statistical metrics, combined with established ccRCC biological characteristics, dictated the stratification of patients into two definitive molecular subtypes. The getMOIC function executed the initial clustering step by deploying ten distinct algorithms (SNF, CIMLR, PINSPlus, NEMO, COCA, moCluster, LRAcluster, ConsensusClustering, IntNMF, and iClusterBayes). The getConsensusMOIC function aggregated these diverse mathematical outputs based on average linkage and Euclidean distance, ultimately yielding a stable, unified patient classification.

### Molecular characterization and subtype stability

Gene Set Variation Analysis (GSVA) was conducted to evaluate the enrichment patterns of treatment-related pathways. Using the RTN R package, we constructed transcriptional regulatory networks mapping 37 chromatin remodeling regulators and 23 target transcription factors associated with activation or repression.

To delineate the TME, the ESTIMATE algorithm was deployed to calculate stromal and immune scores. Methylation levels of tumor-infiltrating lymphocytes (MeTIL) were derived following standard protocols, while the CIBERSORT algorithm quantified the relative abundance of 22 distinct immune cell subsets. To overcome statistical limitations due to limited sample sizes in individual datasets, the E-MTAB-1980 and E-MTAB-3218 datasets were merged to form a unified "Meta-ccRCC" cohort. The reproducibility of the consensus subtypes was strictly validated in a validation group. Additionally, we cross-verified our clustering outcomes against alternative methodologies, specifically partitioning around medoids (PAM) and nearest template prediction (NTP), ensuring consistent structural stability.

### MIME-based prognostic signature generation (101 machine learning frameworks)

The Machine Learning-based Integration of Multiple Evaluators (MIME) framework was utilized to develop a robust prognostic model. MIME capitalizes on ten classical algorithms—including random forest (RSF), least absolute shrinkage and selection operator (Lasso), ridge regression, survival-SVM, GBM, superpc, plsRcox, CoxBoost, StepCox, and elastic net (Enet)—combined with various feature selection techniques (RSF, CoxBoost, StepCox, and Lasso). The workflow encompasses three core stages: data ingestion, iterative model training, and performance visualization. Initially, the framework requires transcriptomic matrices paired with clinical outcomes (survival or therapeutic response) and an input gene set. MIME then automates the training across diverse algorithms to pinpoint core features and forecast clinical trajectories, outputting comprehensive graphical interpretations. Users can then evaluate the predictive accuracy, such as the area under the curve (AUC) or C-index, of the optimized model against externally provided models from existing literature.

In our signature development phase, we first executed univariate Cox regression across the TCGA-KIRC, Meta-ccRCC, and CPTAC-ccRCC cohorts to screen for survival-associated genes (p < 0.05). These candidates were fed into MIME, generating 101 distinct algorithm combinations. To prevent overfitting and guarantee broad applicability, all models were trained strictly on the TCGA-KIRC cohort and independently validated in the Meta-ccRCC and CPTAC-ccRCC sets. The optimal multi-gene signature was selected based on superior cross-cohort predictive performance. A specific prognostic score (CMLS) was computed for all patients, facilitating their categorization into high- or low-CMLS risk groups based on the median score threshold. Survival differences were assessed through Kaplan–Meier curves and log-rank tests utilizing the survminer package (p < 0.05). Additionally, an integrated clinical nomogram was formulated. The predictive reliability of the CMLS model was rigorously confirmed through receiver operating characteristic [16] and calibration curve analyses via the timeROC, survival, and rms R packages.

### Single-cell RNA-seq processing and differential analysis

Downstream scRNA-seq analyses were powered by the Seurat package (v4.3.0). Quality control steps involved the removal of putative doublets via DoubletFinder (v2.0.4) and the exclusion of low-viability cells (defined by < 500 UMIs or mitochondrial gene proportions > 15%). The Harmony integration tool was applied to neutralize sample-specific batch effects. Following the identification of the 2000 most highly variable genes (FindVariableFeatures), PCA was conducted. The first 50 principal components were used to construct a nearest-neighbor graph (FindNeighbors) and define clusters (FindClusters), which were visualized via uniform manifold approximation and projection (UMAP). Clusters were assigned to major cellular lineages based on classic marker genes, including epithelial cells (EPCAM, KRT8/18), fibroblasts (ACTA2, COL1A1/2, DCN), endothelial cells (PECAM1, FLT1, VWF), myeloid populations (CD68, LYZ, C1QB), T/NK cells (CD3D/E, NKG7), B/plasma cells (CD79A/B, IGKC), mast cells (KIT, CPA3, TPSAB1), and proliferating cells (MKI67, CENPF, TOP2A). Differentially expressed genes (DEGs) were identified using the FindAllMarkers function (parameters: only.pos = TRUE, min.pct = 0.25, logfc.threshold = 0.25). Statistical significance was determined utilizing the Wilcoxon rank-sum test with Benjamini–Hochberg adjustment.

### Intercellular communication networks

The CellChat toolkit (v2.2.0) mapped putative signaling cross-talk across distinct cell subpopulations. The CellChatDB.human ligand-receptor interaction database served as the reference to define significant communication pathways (P < 0.05).

### Spatial transcriptomics data analysis

The Seurat package (v4.3.0) processed the ST and Visium spatial matrices. Initial quality control eliminated spots containing fewer than 200 detected genes and discarded genes expressed in fewer than three distinct spots. Following LogVMR normalization, the spatial data underwent dimensionality reduction using the top 30 principal components.

### Immunogenomic profiling and therapeutic response prediction

The IOBR R package was leveraged to quantify an array of established TME signatures—encompassing immune exclusion, immunosuppression, and immunotherapy responsiveness—allowing for a unified assessment of immunological landscapes between CMLS subgroups. We systematically compared the distribution of tumor neoantigen burden (TNB), TMB, and M1 macrophage infiltration across the high- and low-CMLS classifications. To anticipate the efficacy of immune checkpoint blockade, we evaluated delayed response survival and applied the Tumor Immune Dysfunction and Exclusion (TIDE) algorithm, subclass mapping, and the Tumor Immunophenotype [17] profiling method. The robustness of these immunotherapy response predictions was independently verified across three external cohorts (GSE91061, GSE135222, and GSE78220).

### Computational screening for targeted therapeutics

Gene Set Enrichment Analysis (GSEA) mapped the oncogenic pathway activation differences between CMLS strata. For pharmacological screening, the PRISM repurposing repository (1448 compounds, 482 cell lines) and the CTRP v.2.0 dataset (481 compounds, 835 cell lines) provided in vitro drug sensitivity metrics. The AUC quantified compound efficacy; lower values directly indicated stronger drug sensitivity. The K-nearest neighbors (KNN) algorithm imputed missing values for compounds lacking under 20% of their AUC data, while stricter filtering discarded any compounds exceeding this missingness threshold. Anchoring these drug databases to the Broad Institute’s Cancer Cell Line Encyclopedia (CCLE) project enabled direct mapping of CCLE multi-omics data to uncover deeper pharmacogenomic correlations.

### Clinical samples and quantitative real-time polymerase chain reaction (RT-qPCR)

Tissue samples consisted of 17 primary clear cell renal cell carcinomas (ccRCC) and their paired adjacent normal kidney tissues, obtained from patients undergoing surgical resection at the First Affiliated Hospital of Zhengzhou University. Ethical clearance was granted by the institutional review committee (No. 2022-KY-1089-001). Total RNA was extracted from the tissues using Trizol reagent (Takara, Beijing, China), after which complementary DNA was produced using the PrimeScript RT kit equipped with a gDNA removal step. qRT-PCR amplification for the five core CMLS hub genes (PLTP, PRAME, C1QL1, AQP9, and ARL4C) was then performed using SYBR Green reagents (Cowin Biosciences, Jiangsu, China) along with primers sourced from Sangon Biotech (Shanghai, China). GAPDH expression served as the endogenous internal reference for normalization. The relative mRNA abundance of the target genes was calculated and presented as -Δ*C*_*t*_ values.

### Statistical methodology

The R environment (version 4.3.1) handled all computational and statistical operations. Unpaired Student’s t-tests evaluated normally distributed continuous variables, while the Wilcoxon rank-sum test assessed non-parametric data. Multi-group comparisons relied on one-way ANOVA for parametric datasets and Kruskal–Wallis tests for non-parametric distributions. The two-sided Fisher’s exact test compared categorical proportions. To stratify patients, the surv_cutpoint algorithm (from the survminer package) dynamically scanned all potential thresholds to maximize the log-rank statistic, thereby defining the optimal bifurcation point for the CMLS score. Independent cutoff calculations within each dataset successfully mitigated latent computational batch effects. Finally, the limma and MOVICS packages drove the differential expression analyses and consensus multi-omics clustering, respectively.

## Results

### Multi-omics consensus prognosis-related molecular subtypes of ccRCC

**Fig. 1:**
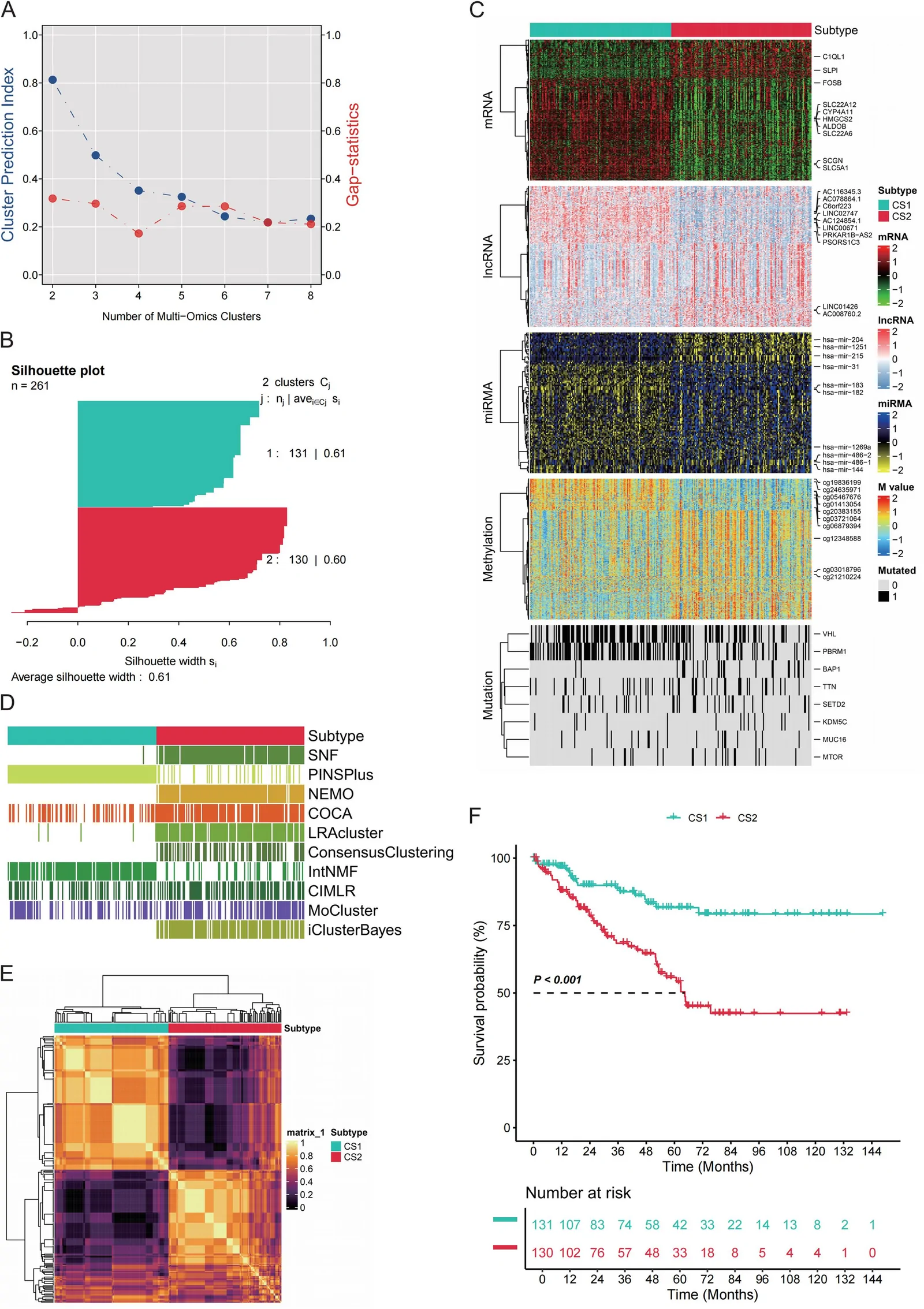
Multi-omics consensus molecular subtyping of ccRCC. **A** Optimal cluster count (k = 2) selection for the multi-omics cohort. **B** Silhouette analysis confirming subgroup stability, with a peak score of 0.61 at k = 2. **C** Heatmap of multi-omics features (mRNAs, lncRNAs, miRNAs, CpG methylation, and somatic mutations) across the two identified subtypes. **D** Comparative clustering outputs for ccRCC patients derived from 10 distinct algorithmic approaches. **E** A consolidated consensus matrix demonstrating the robust division into two subtypes utilizing the 10 integrated algorithms. **F** Kaplan–Meier survival curves comparing clinical outcomes between the identified subtypes

Multi-omics integration mapped the molecular landscape of ccRCC. Cluster evaluation identified two patient subgroups (k = 2) as the ideal stratification (Fig. 1A). Silhouette analysis confirmed this division, showing a peak average score of 0.61 (Fig. 1B). Ten distinct algorithms clustered the patients independently to prevent single-method bias (Fig. 1D). A consensus ensemble approach merged these diverse outputs into a single stable matrix, separating the cohort into two molecular subtypes (Fig. 1E). The multi-omics heatmap displayed clear variations between these groups across mRNA, lncRNA, miRNA expression, CpG methylation, and gene mutations (Fig. 1C). Kaplan–Meier curves highlighted survival differences between the two subtypes (Fig. 1F). This classification links specific molecular features directly to patient prognosis.

### Molecular landscape and external validation of the ccRCC consensus subtypes

**Fig. 2:**
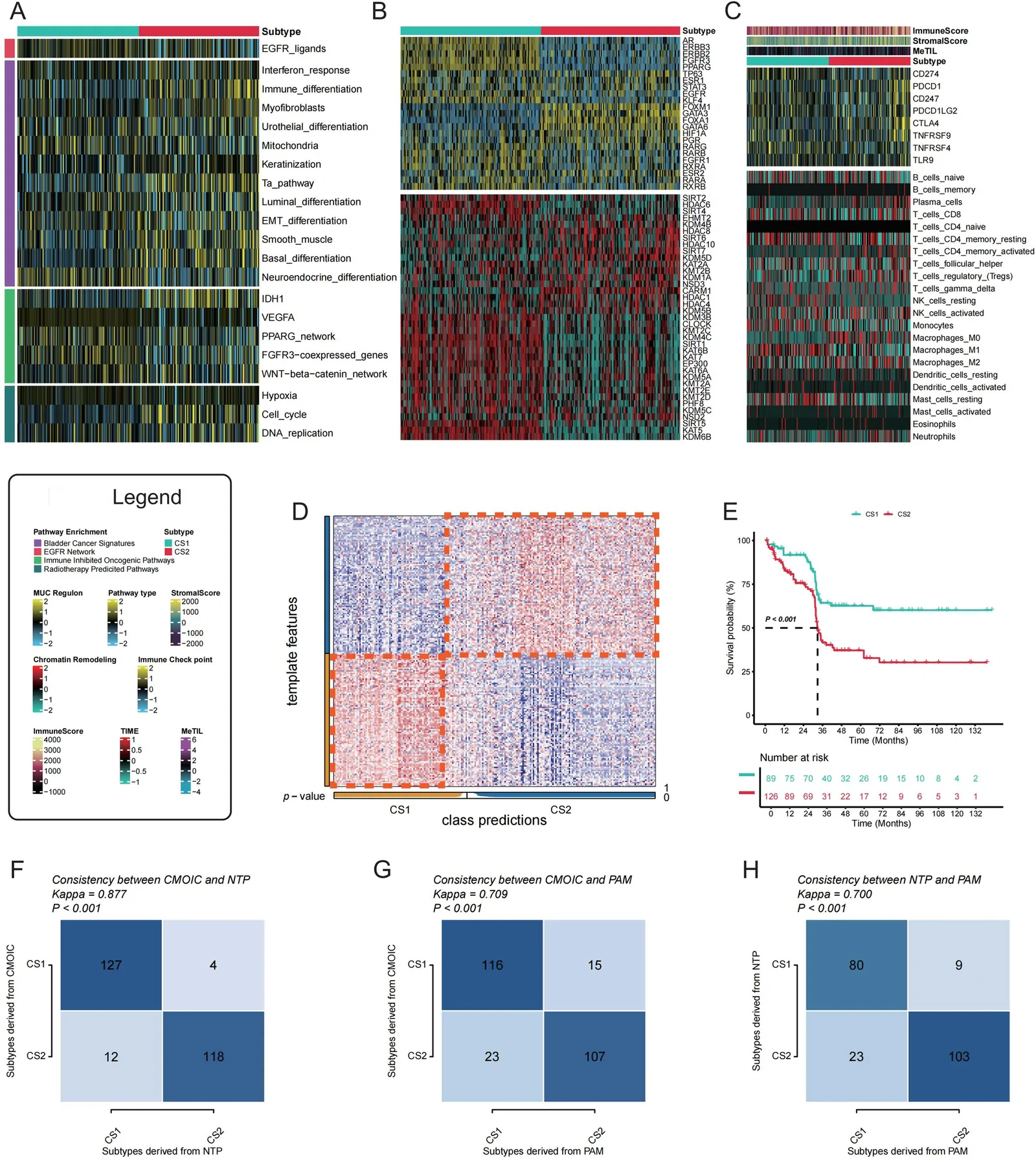
Molecular characterization and external validation of the consensus ccRCC subtypes. **A** Variation in the enrichment of therapy-associated gene signatures between the distinct subgroups. **B** Comparative regulon activity profiles for transcription factors (upper panel) and putative chromatin remodeling regulators (lower panel) across the subtypes. **C** Profiling of the immune microenvironment in the TCGA-KIRC dataset. Upper annotations display stromal/immune enrichment scores and MeTIL (DNA methylation of tumor-infiltrating lymphocytes) levels. Main panels illustrate the differential expression of established immune checkpoints (top) alongside the relative abundance of 22 TME-infiltrating immune cell subsets (bottom). **D** Cross-cohort validation mapping of the established molecular subtypes within the Meta-ccRCC dataset. **E** Prognostic divergence of the identified subtypes confirmed in the Meta-ccRCC validation cohort. **F**–**H** Concordance evaluations demonstrating the structural stability of the clustering outcomes when compared against NTP classification (**F**), PAM classification (**G**), and the cross-comparison between NTP and PAM methodologies (**H**) within the Meta-ccRCC cohort

The two ccRCC subtypes showed different enrichment patterns for treatment-related signatures and molecular pathways (Fig. 2A). Regulon activity profiles for transcription factors and chromatin remodeling regulators also varied between the groups (Fig. 2B). These regulatory differences indicate that epigenetic networks drive specific molecular phenotypes. Immune profiling of the TCGA-KIRC cohort detailed stromal and immune enrichment scores alongside MeTIL levels (Fig. 2C). The subtypes displayed divergent expression levels of classic immune checkpoint genes and varied infiltration of 22 immune cell populations. These patterns suggest the subtypes represent different states of immune activation. For external validation, merging E-MTAB-1980 and E-MTAB-3218 created the Meta-ccRCC cohort. Initial PCA showed batch effects from dataset-specific clustering (Figure S1A). Transcriptomic harmonization removed these technical variations, and post-correction PCA confirmed successful data integration (Figure S1B). The NTP algorithm assigned each sample in this meta-cohort to a molecular subtype using template features (Fig. 2D). Survival analysis in this group confirmed the prognostic difference between the subtypes (P < 0.001, Fig. 2E). Comparing the primary clustering results with NTP (Kappa = 0.877, P < 0.001; Fig. 2F) and PAM classifiers (Kappa = 0.709, P < 0.001; Fig. 2G) showed consistent structural alignment. NTP and PAM classifications also matched closely within the Meta-ccRCC cohort (Fig. 2H).

### Development and validation of the CMLS

**Fig. 3:**
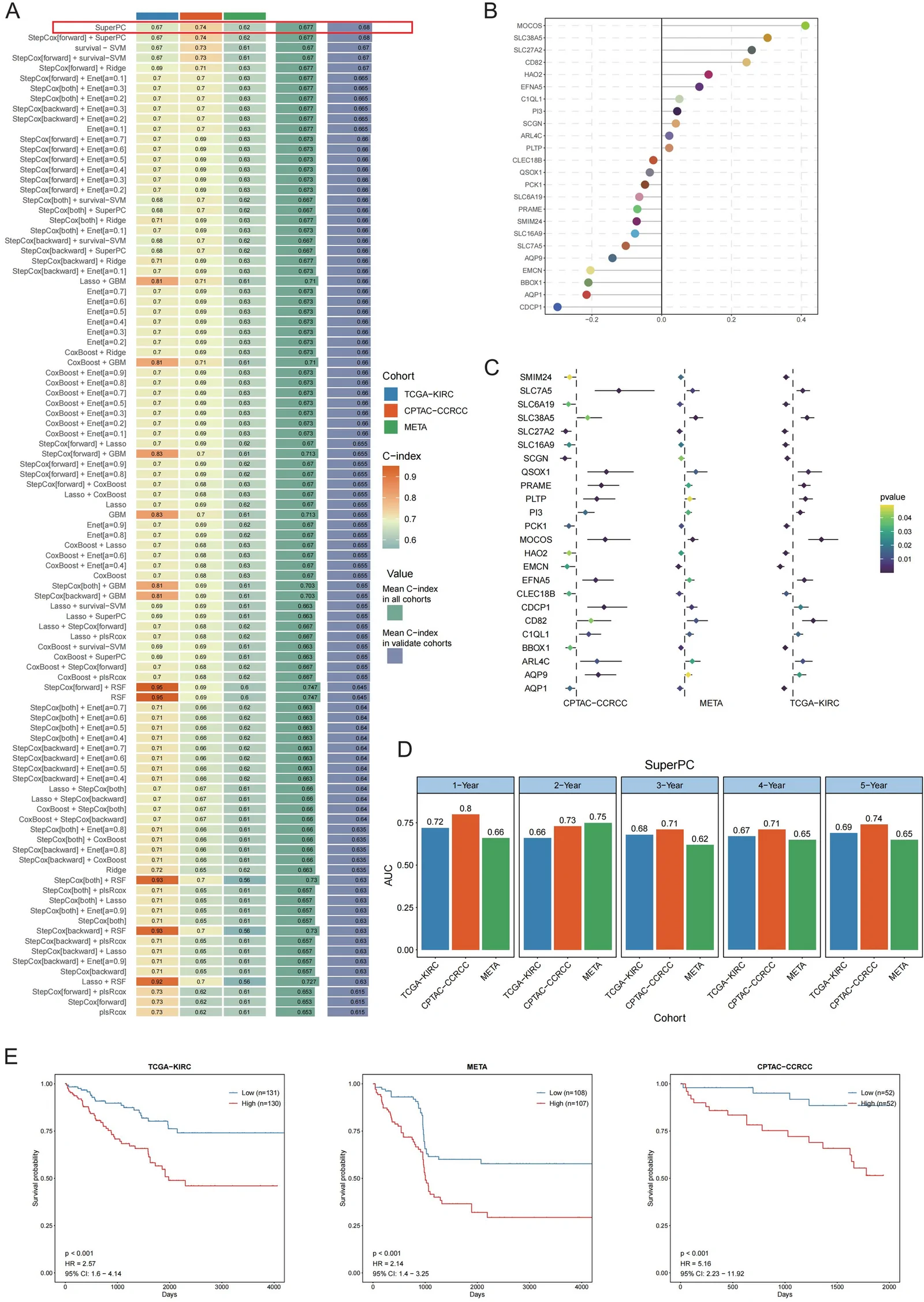
Development and clinical validation of the Consensus Machine Learning-driven Signature (CMLS). **A** Performance ranking of 101 distinct machine-learning algorithmic combinations. Models were evaluated based on their C-index across the TCGA-KIRC, CPTAC-ccRCC, and Meta-ccRCC cohorts, and ordered by average validation performance. **B** Identification of core prognostic hub genes utilizing the optimal SuperPC algorithm. **C** Forest plots detailing the univariate Cox regression statistics for the identified hub genes across both training and external validation sets. **D** Time-dependent ROC curves illustrating the predictive accuracy (AUC metrics) of the SuperPC-derived model for 1- to 5-year overall survival across all evaluated cohorts. **E** Kaplan–Meier survival trajectories comparing high- versus low-CMLS risk groups within the TCGA-KIRC, Meta-ccRCC, and CPTAC-ccRCC patient populations

Screening 101 machine learning combinations evaluated their predictive power to construct a consensus prognostic signature. The C-index quantified each model’s performance across the TCGA-KIRC, CPTAC-ccRCC, and Meta-ccRCC cohorts. Ranking these models by their average C-index in the validation sets identified the best computational approach (Fig. 3A). The SuperPC algorithm delivered the highest predictive stability. This algorithm extracted the core hub genes to build the signature (Fig. 3B). Univariate Cox regression across all cohorts confirmed these genes linked directly to patient survival (Fig. 3C). Time-dependent ROC curves tracked the model’s accuracy over extended follow-ups. The signature produced high AUC values for 1-, 2-, 3-, 4-, and 5-year survival rates across the three cohorts (Fig. 3D). Each patient received a specific CMLS score. The median value split the populations into high- and low-CMLS groups. Kaplan–Meier curves showed that high CMLS scores correlated with shorter overall survival across the TCGA-KIRC, Meta-ccRCC, and CPTAC-ccRCC datasets (Fig. 3E). A meta-analysis of univariate Cox models from these cohorts verified the CMLS as an independent risk factor (HR = 2.48, P < 0.001; Figure S2A). Multivariate Cox regression proved the CMLS maintained its prognostic value even when adjusted for age, tumor grade, and clinical stage (Figure S2B, S2C). A clinical nomogram combined the CMLS with these clinical features to estimate 1-, 3-, and 5-year survival probabilities (Figure S2F). Calibration curves showed tight alignment between the nomogram predictions and actual survival (Figure S2D). Decision Curve Analysis (DCA) confirmed the net clinical benefit of the model across various threshold probabilities (Figure S2E). Time-dependent C-index tracking verified the sustained accuracy of this combined framework (Figure S2G).

### Single-cell transcriptomic atlas decodes the cell type-specific expression patterns of the CMLS

**Fig. 4:**
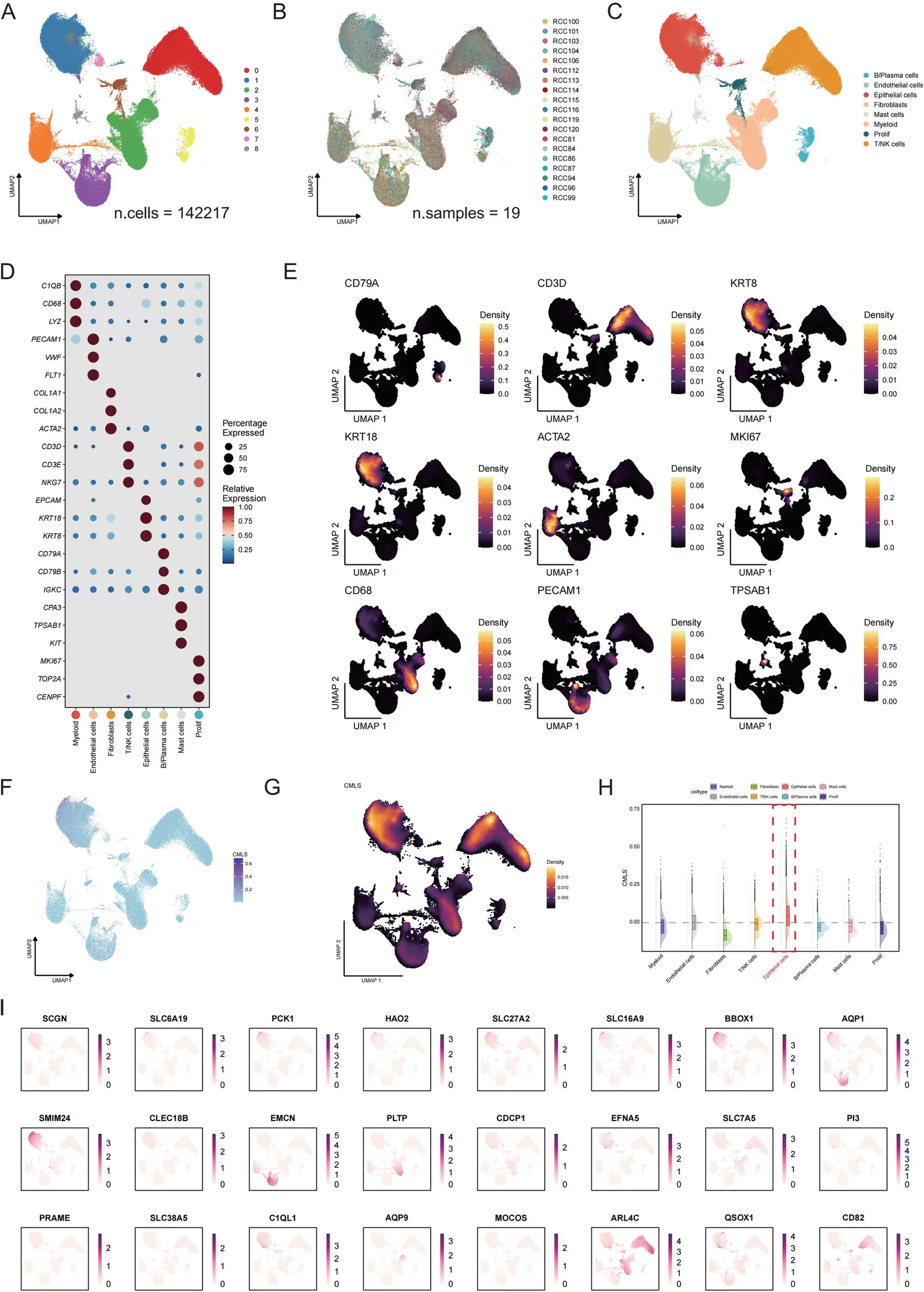
High-resolution single-cell transcriptomic mapping of CMLS expression patterns. **A** UMAP projection of 142,217 cells grouped into 10 Seurat clusters. **B** Post-integration UMAP embedding highlighting the successful removal of technical batch effects across the 19 biological samples. **C** Annotation of major cellular lineages guided by canonical marker profiling, encompassing B/plasma cells, T/NK cells, epithelial cells, endothelial cells, fibroblasts, myeloid cells, mast cells, and proliferating populations. **D** Dot plot characterizing the relative expression levels of lineage-defining marker genes across the annotated cellular subsets. **E** Density distributions illustrating the localized expression intensities of these canonical markers. **F** Single-cell feature plot mapping the aggregated spatial expression of the CMLS signature within the TME. **G** Density mapping reflecting the global distribution of CMLS scores at the single-cell resolution. **H** Quantitative breakdown of CMLS enrichment across the distinct annotated cell lineages. **I** Individual feature plots delineating the specific cellular localization of the 24 constituent module genes comprising the CMLS

Quality control filters eliminated technical artifacts, retaining 142,217 cells from 19 scRNA-seq samples. The Harmony algorithm corrected sample-specific batch effects across this dataset. UMAP visualizations then separated these integrated cells into 10 distinct clusters (Fig. 4A, B). Canonical marker tracking assigned these clusters to eight biological lineages: B/plasma cells, endothelial cells, epithelial cells, fibroblasts, mast cells, myeloid cells, proliferating cells, and T/NK cells (Fig. 4C). Dot and density plots verified the expression trajectories of these specific markers (Fig. 4D, E).

Mapping the CMLS score onto this annotated landscape revealed uneven distribution across different TME niches (Fig. 4F, G). Quantitative lineage analysis traced the highest risk scores to specific cell subpopulations (Fig. 4H). The 24 module genes forming the CMLS also demonstrated localized, population-specific expression rather than widespread activation (Fig. 4I). This mapping confirms that bulk transcriptomic risk originates from distinct cellular sources.

### Intercellular communication rewiring and high-resolution spatial mapping dictated by the CMLS

**Fig. 5:**
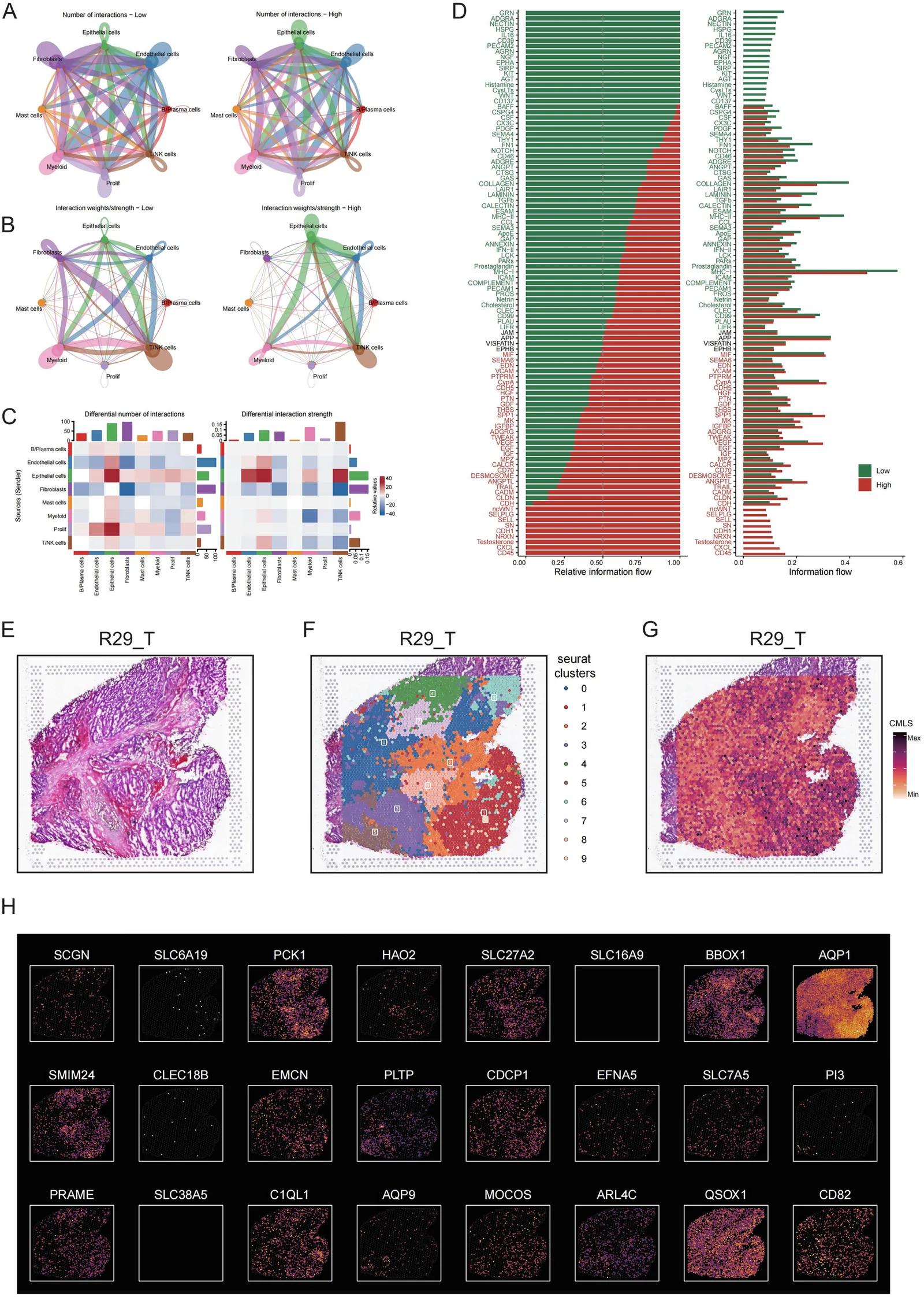
Spatial transcriptomic deconvolution and TME interactome rewiring driven by the CMLS. **A** Circos diagrams illustrating the topological shifts in the total quantity of intercellular ligand-receptor crosstalk between the high- and low-CMLS strata. **B** Visual representation of the varying communication probabilities and interaction strengths among cellular lineages, stratified by CMLS risk. **C** Differential heatmaps quantifying the net alterations in both the volume and intensity of cell–cell communication networks when comparing high- versus low-CMLS conditions. **D** In-depth profiling of specific signaling axes, highlighting disparities in pathway activation strength and communication frequency dictated by the CMLS status. **E** H&E staining of the ccRCC tissue section (R29_T). **F** Spatial clustering of tumor spots using Seurat. **G** Spatial distribution of CMLS scores across the tissue section. **H** High-definition spatial mapping demonstrating the localized expression gradients of the 24 individual CMLS module genes across the microenvironmental spots

Ligand-receptor network analysis linked CMLS expression to intercellular crosstalk. Circos plots displayed large variations in communication quantity and strength between the high- and low-CMLS groups (Fig. 5A, B). Heatmaps tracked these interaction shifts directly to the dense signaling among epithelial cells, fibroblasts, and immune populations (Fig. 5C). High CMLS scores altered the information flow within extracellular matrix networks (COLLAGEN, LAMININ) and immune-regulatory axes (CXCL, CD45) (Fig. 5D).

ST data localized these interactions within intact tissue. H&E staining and spatial clustering of section R29_T mapped the structural niches of the tumor (Fig. 5E, F). Projecting the aggregated CMLS score onto this section anchored the risk signal to defined anatomical zones (Fig. 5G). The 24 module genes followed similar spatial gradients across these microenvironmental spots (Fig. 5H). This physical mapping aligns with the scRNA-seq data, proving the signature reflects actual tissue architecture.

### Distinct tumor immune microenvironment landscapes and immunotherapeutic vulnerabilities dictated by the CMLS

**Fig. 6:**
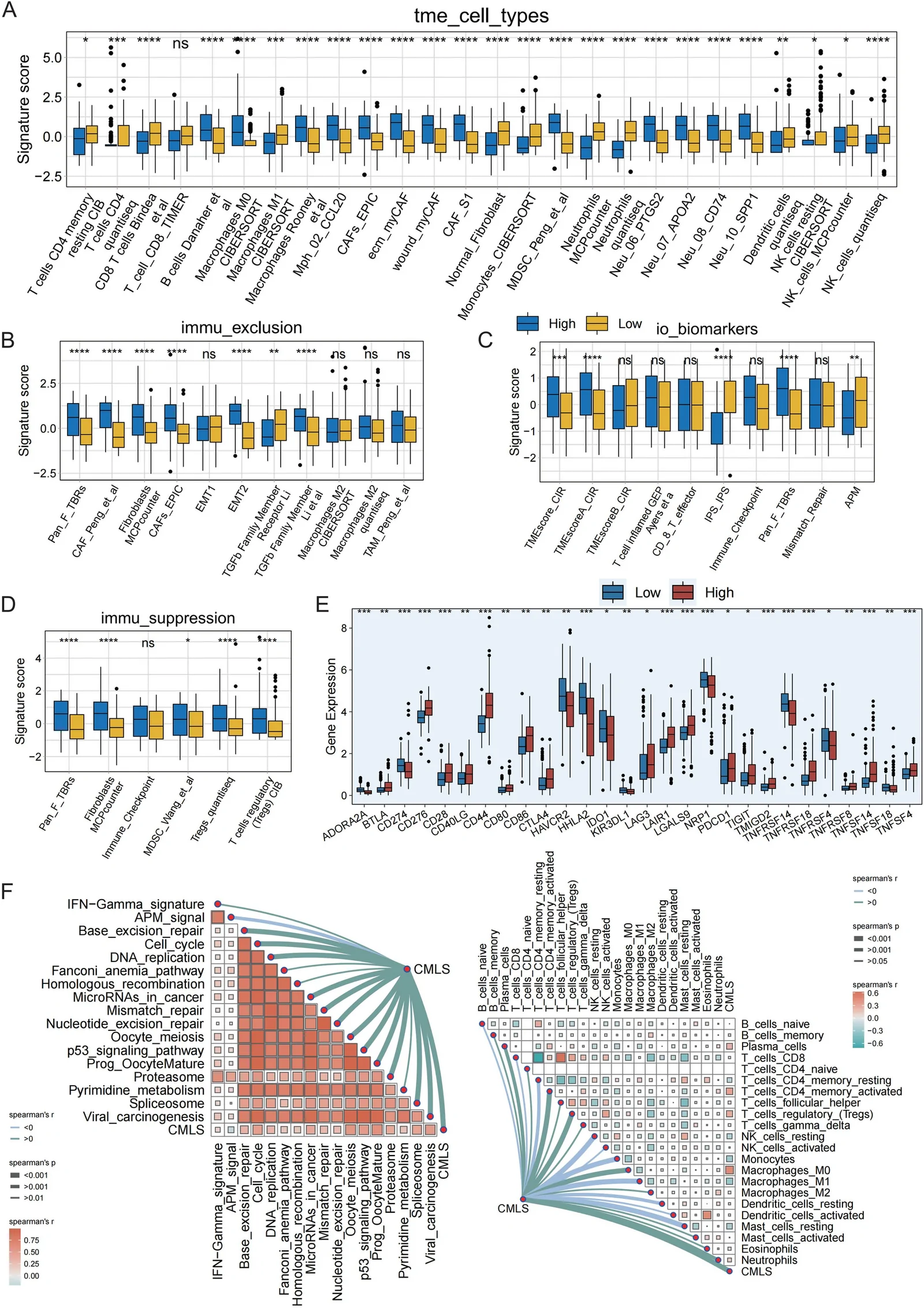
Immunological landscapes and microenvironmental architectures stratified by CMLS risk. **A**–**D** Comparative quantification of established TME immunologic profiles between high- and low-CMLS patients, detailing the enrichment of specific immune cell signatures (**A**), markers of immune exclusion (**B**), established indicators of immunotherapy response (**C**), and pathways governing immunosuppression (**D**). **E** Differential expression profiles of critical immune checkpoint molecules evaluated across the disparate CMLS classifications. **F** Comprehensive correlative mapping linking the CMLS risk score with predictive indices for immune checkpoint blockade efficacy and the relative infiltration of 22 specific immune cell subsets (derived via CIBERSORT)

Evaluating established immunologic panels exposed distinct TME features separating the high- and low-CMLS strata. Quantitative tests showed varying enrichment of specific immune cell types (Fig. 6A) alongside differences in standard immunotherapy biomarkers (Fig. 6C). High-CMLS tumors physically displayed patterns of immune exclusion (Fig. 6B) and active immune suppression (Fig. 6D). Box plots tracked the expression of classic checkpoint genes—CD274 (PD-L1), PDCD1 (PD-1), and CTLA4—across the patient groups (Fig. 6E). This checkpoint variation dictates the basal immunomodulatory capacity of the respective subtypes. Correlation analysis linked the CMLS score directly to the CIBERSORT-quantified infiltration of 22 immune cell subsets and predictive ICB response traits (Fig. 6F). The genetic risk signature therefore directly tracks with immune cell recruitment and microenvironmental states.

### The CMLS robustly dictates immunotherapeutic efficacy and clinical outcomes

**Fig. 7:**
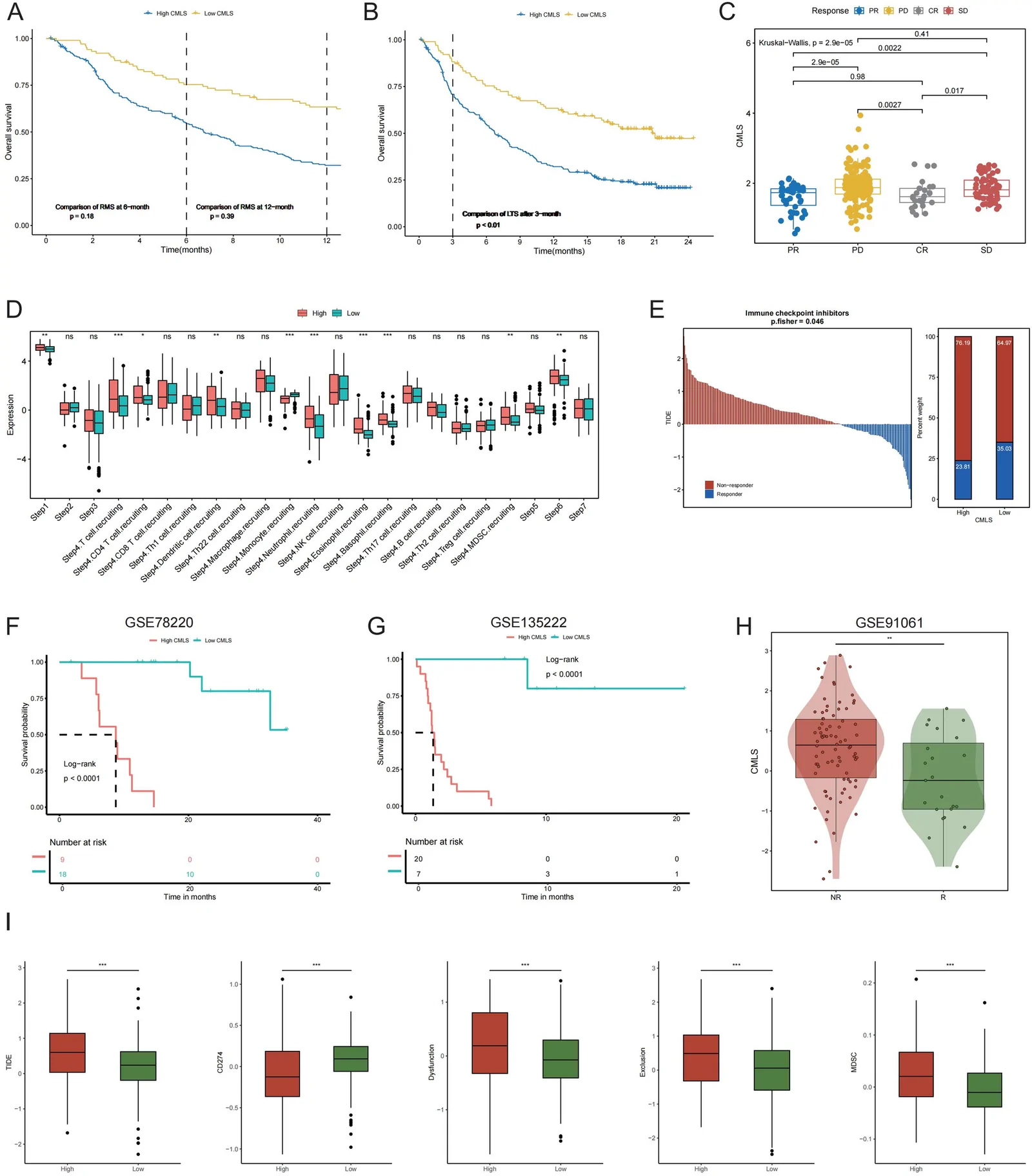
Predictive utility of the CMLS in determining systemic immunotherapeutic efficacy. **A** Evaluation of RMS disparities at 6- and 12-month post-treatment intervals, contrasting the high- and low-CMLS populations. **B** Assessment of LTS advantages emerging after an initial 3-month therapeutic window. **C** Stratification of the CMLS risk score across patients exhibiting divergent clinical responses to immunotherapy. **D** Dissection of the cancer-immunity cycle using the TIP framework, highlighting varying activation states at each progressive immunological step between the distinct risk strata. **E** Estimated probabilities of therapeutic response derived from the TIDE computational algorithm. **F**–**G** Independent prognostic validation demonstrating survival divergence between CMLS groups in the external ICB-treated GSE78220 (**F**) and GSE135222 (**G**) cohorts. **H** Correlation of CMLS levels with documented clinical response categories within the GSE91061 dataset. **I** Quantitative comparison of overall TIDE scores between patients harboring high versus low CMLS

The CMLS score directly forecasted clinical responses to systemic ICB therapy. Patients in the low-score group achieved longer restricted mean survival (RMS) at 6 and 12 months (Fig. 7A) alongside extended long-term survival (LTS) (Fig. 7B). This survival benefit aligned tightly with actual treatment outcomes. Individuals reaching complete or partial response (CR/PR) presented much lower risk scores than non-responders experiencing stable or progressive disease (SD/PD) (Kruskal–Wallis test, P = 2.9e-05; Fig. 7C). The genetic signature therefore accurately detects baseline sensitivity to immune interventions. The TIP framework mapped the dynamic phases of the cancer-immunity cycle to explain these varying clinical outcomes. Distinct risk groups showed different activation levels across anti-tumor immune steps, spanning initial antigen priming, immune cell recruitment, and target eradication (Fig. 7D). The TIDE algorithm provided additional computational evidence for this functional breakdown. High risk scores linked directly to elevated TIDE metrics, reflecting severe baseline T cell dysfunction and active immune evasion (Fig. 7I). This localized immune exclusion drastically reduced the predicted probability of a positive immunotherapy response (Fig. 7E). Both single-cell and spatial data support this conclusion, confirming the signature actively shapes an immune-depleted TME.

Evaluating the CMLS alongside conventional biomarkers like TMB and TNB clarified its independent clinical value. Linear regression confirmed a positive correlation with TIDE scores (Figure S3E), while cell infiltration data revealed a severe depletion of anti-tumorigenic M1 macrophages in the high-risk group (Figure S3C, S3D). The overall distribution of TMB and TNB remained consistent across risk groups (Figure S3A, S3B). This lack of variance proves the multi-gene model captures biological dimensions completely separate from macroscopic mutational loads. Combining the new signature with TMB, TNB, or M1 abundance created a highly refined prognostic matrix. Survival curves proved that low-risk patients maintained longer life expectancies irrespective of their baseline mutational burden or macrophage status (Figure S3F–H). The new metric effectively supplements standard genomic indicators to improve patient stratification.

Independent transcriptomic cohorts receiving targeted ICB tested the broader applicability of this predictive model. Survival analyses in the external GSE78220 and GSE135222 datasets confirmed that lower scores lead to extended overall survival (P < 0.0001; Fig. 7F, G). The GSE91061 dataset linked the signature directly to documented clinical outcomes, showing higher response rates in the low-risk population (Fig. 7H). These multi-cohort tests validate the model as a practical tool to bypass immune resistance in the clinic.

### Identification of therapeutic vulnerabilities and potential pharmacological interventions dictated by the CMLS

**Fig. 8:**
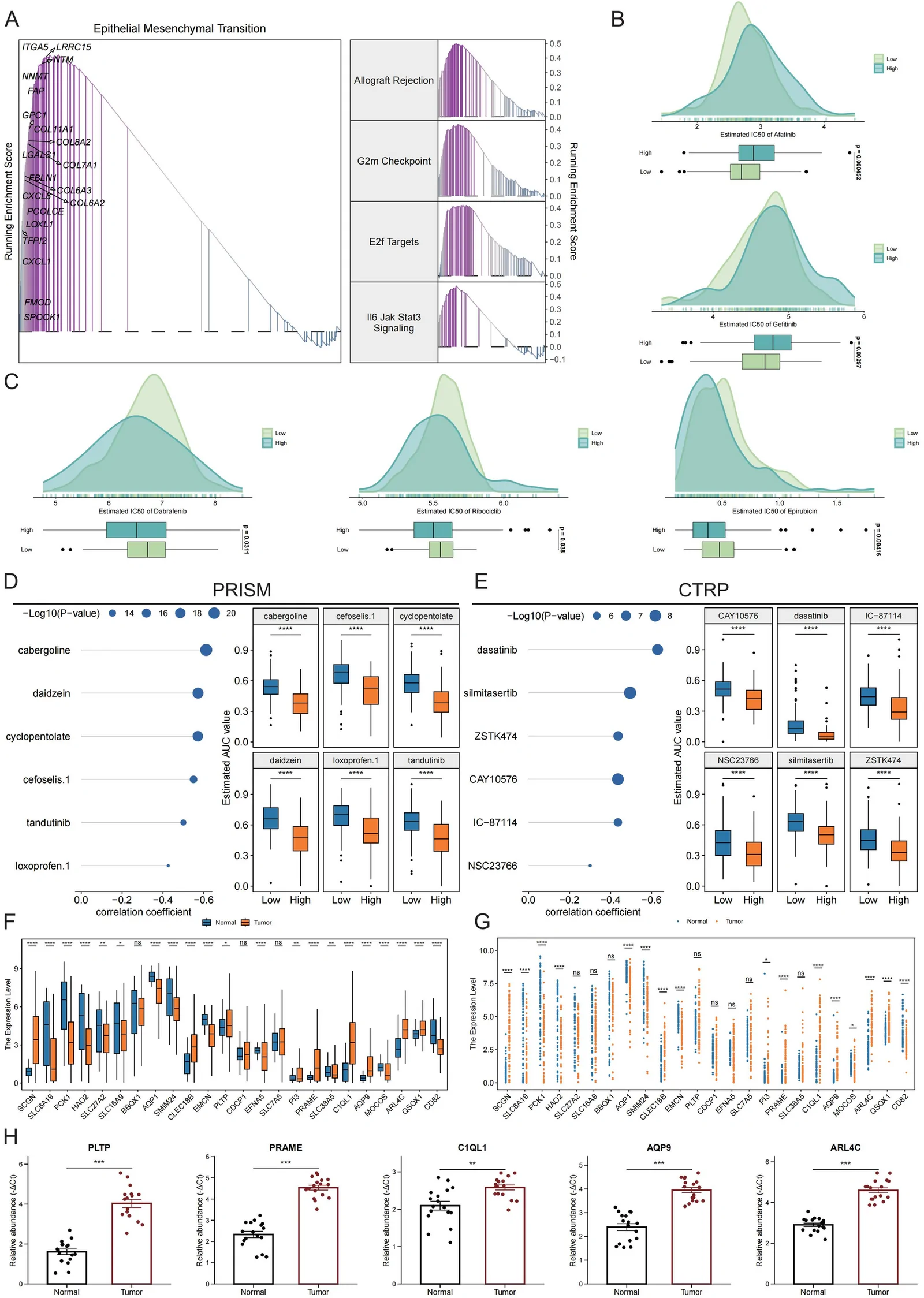
Pharmacogenomic vulnerabilities and targeted therapeutic candidate screening based on CMLS. **A** Identification of hyperactive oncogenic networks within the high-CMLS subset utilizing GSEA. **B** GDSC-derived sensitivity metrics showing resistance to EGFR inhibitors (afatinib and gefitinib) in high-risk patients. **C** Sensitivity of the high-CMLS population to targeted agents (dabrafenib, ribociclib, and epirubicin) based on GDSC profiles. **D**–**E** Correlation and differential efficacy of novel pharmacological candidates from PRISM (**D**) and CTRP (**E**) databases. **F**–**G** Unpaired (**F**) and paired (**G**) expression differences of the 24 CMLS module genes between ccRCC and adjacent normal tissues in the TCGA-KIRC cohort. (*p < 0.05, **p < 0.01, ***p < 0.001). **H** Experimental validation of the five core CMLS hub genes (PLTP, PRAME, C1QL1, AQP9, and ARL4C) via RT-qPCR in an independent cohort of 17 paired ccRCC and adjacent normal tissues. The relative mRNA abundance is calculated using the -Δ*C*_*t*_ method with GAPDH serving as the internal control. (*p < 0.05, **p < 0.01, ***p < 0.001)

GSEA mapped the specific oncogenic networks causing poor clinical outcomes in the high-risk group. This population showed heavy enrichment for EMT, G2M checkpoint, E2F targets, and IL6/JAK/STAT3 signaling cascades (Fig. 8A). Overactive transcriptional pathways directly drive the aggressive biological behavior of these tumors.

The GDSC database supplied pharmacogenomic profiles to translate these molecular features into targeted treatment strategies. Tumors with elevated scores resisted typical epidermal growth factor receptor (EGFR) inhibitors, presenting higher estimated IC50 values for afatinib and gefitinib (Fig. 8B). This identical high-risk population maintained strong sensitivity to alternative agents like dabrafenib, ribociclib, and epirubicin (Fig. 8C). The risk metric effectively separates baseline drug responses to locate treatment windows for refractory cancers.

The PRISM and CTRP datasets provided in silico drug screening data to expand potential treatment options. Differential sensitivity testing identified novel candidates, such as cabergoline and dasatinib, which deliver targeted cytotoxic effects against the high-risk phenotype (Fig. 8D, E). Expression analysis of the 24 module genes confirmed the biological foundation of the signature. Both unpaired and paired transcriptomic analyses within the TCGA-KIRC cohort consistently elucidated profound expression dysregulation of these core features in malignant ccRCC tissues compared to adjacent normal counterparts (Fig. 8F, G). To validate these in silico transcriptomic findings within a real-world clinical setting, we performed RT-qPCR on an independent, in-house cohort comprising 17 paired ccRCC and adjacent normal tissues. Consistent with the public dataset predictions, the relative abundance (-Δ*C*_*t*_) of five core CMLS hub genes—PLTP, PRAME, C1QL1, AQP9, and ARL4C—was significantly upregulated in the malignant tissues (Fig. 8H). Ultimately, this comprehensive pharmacogenomic profiling and experimental validation underscore the CMLS as a robust translational framework for tailoring precision pharmacological interventions.

## Discussion

The intra- and inter-tumoral heterogeneity of ccRCC severely undermines the predictive precision of conventional single-omics biomarkers [18, 19]. To bridge the translational gap between macroscopic risk stratification and microscopic TME architecture, we developed the CMLS by integrating multi-omics clustering with a massive ensemble of 101 machine-learning algorithms. Unlike traditional bulk-derived models, the CMLS provides a spatially resolved diagnostic paradigm, demonstrating that patient prognosis and immunotherapeutic vulnerability are fundamentally orchestrated by specific spatial interactions and intercellular rewiring within the TME [20].

Molecular subtyping clarifies ccRCC pathogenesis. The multi-omics classification separates patients into groups driven by distinct epigenetic reprogramming and transcription factor networks. These specific biological divisions align with the metabolic and immune-desert phenotypes observed in earlier ccRCC cohorts [21, 22]. The molecular clusters also display varying MeTIL levels [23]. Anchoring the CMLS model to these solid biological boundaries ensures the predictive metric reflects actual genomic and epigenetic alterations rather than random computational noise.

Standard bulk sequencing averages transcriptomic signals, hiding the physical cellular structure of the tumor [24]. Mapping the CMLS onto a 140,000-cell atlas overcame this technical limitation. The single-cell projection traced the exact origin of the risk signal directly to malignant epithelial and fibroblast compartments [25]. Intercellular interactome mapping revealed that an elevated CMLS is intrinsically linked to topological TME rewiring, characterized by the hyperactivation of EMT and IL6/JAK/STAT3 signaling within cancer-associated fibroblasts (CAFs) [26, 27]. Integrated with our spatial transcriptomic mapping, these findings support a highly localized microenvironmental mechanism: under CMLS-high conditions, matrix-remodeling CAFs and specialized myeloid subsets cooperate to construct a putative immune-excluded niche [28, 29]. This spatial architecture actively restricts CD8 + T cell infiltration into the tumor core, providing high-resolution multi-omics evidence for recent findings on CAF-mediated immunosuppression [20].

The ultimate clinical utility of any prognostic model hinges on its capacity to guide immunotherapeutic interventions. While existing metrics like TMB [7, 30] and established signatures (e.g., JAVELIN Renal 101, IMmotion151) [8, 31] offer valuable insights, the CMLS captures distinct, orthogonal biological features of immune evasion. Our analyses demonstrate that patients in the high-CMLS stratum exhibit a profound depletion of anti-tumorigenic M1 macrophages [32] and a systemic breakdown across the cancer-immunity cycle [33]. Notably, even within the TMB-low subgroup, the CMLS effectively identifies states of severe T-cell exhaustion [34], highlighting its potential as a robust biomarker for combinatorial patient stratification. Validated across independent multi-center cohorts (e.g., GSE78220, GSE135222) [35, 36], the CMLS robustly identifies patients who are most likely to derive long-term survival benefits from systemic ICB, effectively filling predictive gaps left by isolated genomic markers [37].

Addressing the dismal clinical outcomes of the high-risk population necessitates the identification of actionable pharmacological targets. The hyperactivation of malignancy-associated networks, particularly the EMT and IL6 cascades, not only drives an aggressive phenotype in high-CMLS tumors but also exposes distinct pharmacogenomic vulnerabilities [38, 39]. Computational screening across the GDSC, PRISM, and CTRP datasets isolated specific drug response patterns. High-risk tumors resisted standard EGFR inhibitors like gefitinib [40]. These identical resistant populations maintained clear sensitivity to alternative targeted agents, including dabrafenib, ribociclib, and dasatinib [41, 42]. Identifying these drug sensitivities offers a practical method to build CMLS-guided combination therapies and bypass baseline treatment resistance.

The current study design carries specific analytical and biological limitations. Relying on validation set scores to select the SuperPC algorithm creates a circularity issue, potentially skewing the true generalization of the model [43]. Merging data from different sequencing platforms also leaves behind unavoidable technical batch effects [44]. Biologically, while the ST deconvolution is constrained by a single-sample origin, we successfully mitigated the limitations of purely in silico predictions by experimentally validating the significant dysregulation of five core CMLS hub genes in an independent clinical cohort via RT-qPCR. Although these physical tests bridge the gap between macroscopic risk and tissue-level expression, confirming the extensive spatial architectures and high-throughput drug targets will necessitate larger multi-center experimental cohorts and in vivo functional models [45]. Finally, given the retrospective nature of the training datasets, future prospective clinical trials in ICB-treated populations are required to standardize predictive thresholds prior to adapting this signature into routine clinical assays (e.g., qPCR or NanoString panels) for formalin-fixed paraffin-embedded (FFPE) samples.

## Conclusions

The CMLS functions as a direct, quantifiable measure of the immune-excluded microenvironment in ccRCC. Projecting these macroscopic risk scores onto single-cell and spatial atlases isolated the exact cellular populations driving treatment resistance. This biological anchoring explains why the signature predicts immunotherapy outcomes more reliably than traditional indicators like TMB. Beyond immunotherapy stratification, the predictive model flags specific targeted drug sensitivities for high-risk patients. This multi-scale pipeline thereby converts raw transcriptomic data into clear clinical decisions, offering a practical analytical approach for managing complex malignancies.

## Supplementary Information

Below is the link to the electronic supplementary material.

Supplementary Material 1.

Supplementary Material 2.

Supplementary Material 3.

## Data Availability

The publicly available datasets analyzed in this study (including TCGA-KIRC, CPTAC-ccRCC, and Meta-ccRCC) can be accessed through their respective public repositories (e.g., The Cancer Genome Atlas and Gene Expression Omnibus). The datasets supporting the conclusions of this article are included within the article and its additional files.

## Abbreviations

AUC: Area under the curve
CAFs: Cancer-associated fibroblasts
CCLE: Cancer Cell Line Encyclopedia
ccRCC: Clear cell renal cell carcinoma
CMLS: Consensus Machine Learning-driven Signature
CPI: Clustering Prediction Index
CR/PR: Complete or partial response
DCA: Decision Curve Analysis
DEGs: Differentially expressed genes
EGFR: Epidermal growth factor receptor
EMT: Epithelial-Mesenchymal Transition
Enet: Elastic net
FFPE: Formalin-fixed paraffin-embedded
GSEA: Gene Set Enrichment Analysis
GSVA: Gene Set Variation Analysis
ICB: Immune checkpoint blockade
KNN: K-nearest neighbors
Lasso: Least absolute shrinkage and selection operator
LTS: Long-term survival
MeTIL: Methylation levels of tumor-infiltrating lymphocytes
MIME: Machine Learning-based Integration of Multiple Evaluators
NTP: Nearest template prediction
PAM: Partitioning around medoids
PCA: Principal component analysis
RMS: Restricted mean survival
ROC: Receiver operating characteristic
RSF: Random forest
scRNA-seq: Single-cell RNA sequencing
SD/PD: Stable or progressive disease
ST: Spatial transcriptomics
TIDE: Tumor Immune Dysfunction and Exclusion
TIP: Tumor Immunophenotype
TMB: Tumor mutational burden
TME: Tumor microenvironment
TNB: Tumor neoantigen burden
TPM: Transcripts per million
UMAP: Uniform manifold approximation and projection
