# Supplementary material for: Machine learning and multi-omics clustering to map cellular rewiring and immune evasion in ccRCC
Source: Hum Genomics. 2026 Sep 15;20:138. doi: 10.1186/s40246-026-01024-8 (PMC13576295; doi:10.1186/s40246-026-01024-8)
Supplement: Supplementary file 1 [file 40246_2026_1024_MOESM1_ESM.pdf]

郑州大学第一附属医院
科研项目伦理审查委员会审查证明

伦理审查编号：2022-KY-1089-001

项目	转录因子 LMX1B 抑制肾癌细胞增殖和迁移的机制研究		
科室（病区）	郑州大学第一附属医院泌尿外科二病区		
审查方式	快速审查	项目负责人	杨锦建
审查文件清单	初始审查文件：<br>1. 伦理审查申请表<br>2. 研究方案（版本号:v1.0 版本日期:2021-04-06）<br>3. 知情同意书（版本号:v1.0 版本日期:2021-04-06）<br>4. 主要研究者履历<br>5. 科学研究学术评价受理单<br>6. 科研伦理快审登记表<br>7. 答辩 ppt<br>8. 医学研究登记备案系统截图		
审查意见	本项目已通过伦理委员会审查。		

注：

（1）该审查证明仅用于科研人员临床科研目的，不用作产品上市、注册等商业目的。科研人员应在医学研究登记备案平台完成所有流程后再开展研究。

（2）带有“KY”编号的伦理审查证明，为正式的科研伦理审查证明。

（3）带有“SB”编号的伦理审查证明，仅供申报国家级、省市级等课题使用，不得在郑州大学第一附属医院执业范围内开展研究，不得用于发表文章。

郑州大学第一附属医院临床试验伦理委员会

2022 年 09 月 20 日
