# Supplementary material for: Machine learning and multi-omics clustering to map cellular rewiring and immune evasion in ccRCC
Source: Hum Genomics. 2026 Sep 15;20:138. doi: 10.1186/s40246-026-01024-8 (PMC13576295; doi:10.1186/s40246-026-01024-8)
Supplement: Supplementary file 2 [file 40246_2026_1024_MOESM2_ESM.pdf]

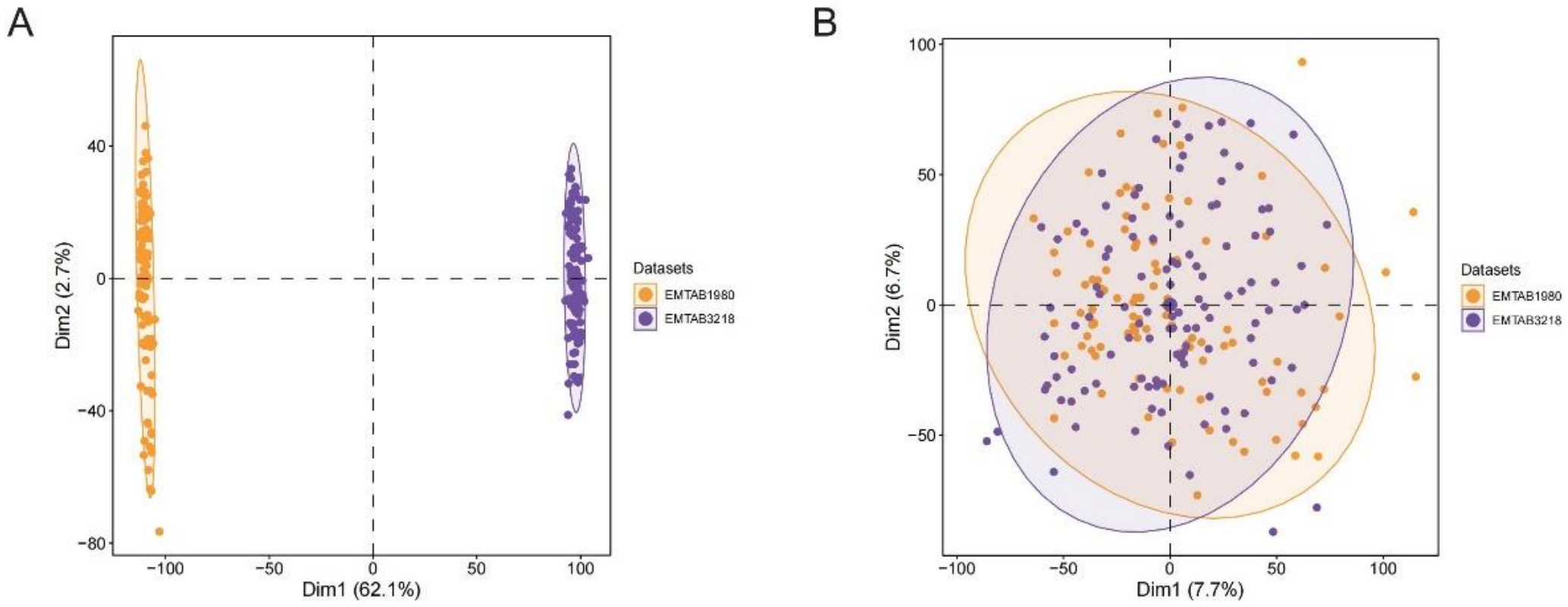

Figure S1. Principal component analysis (PCA) validating the harmonization of the Meta-ccRCC composite cohort.

(A) Multidimensional projection revealing dataset-specific clustering prior to harmonization.

(B) Post-correction visualization confirming the successful elimination of technical batch effects.

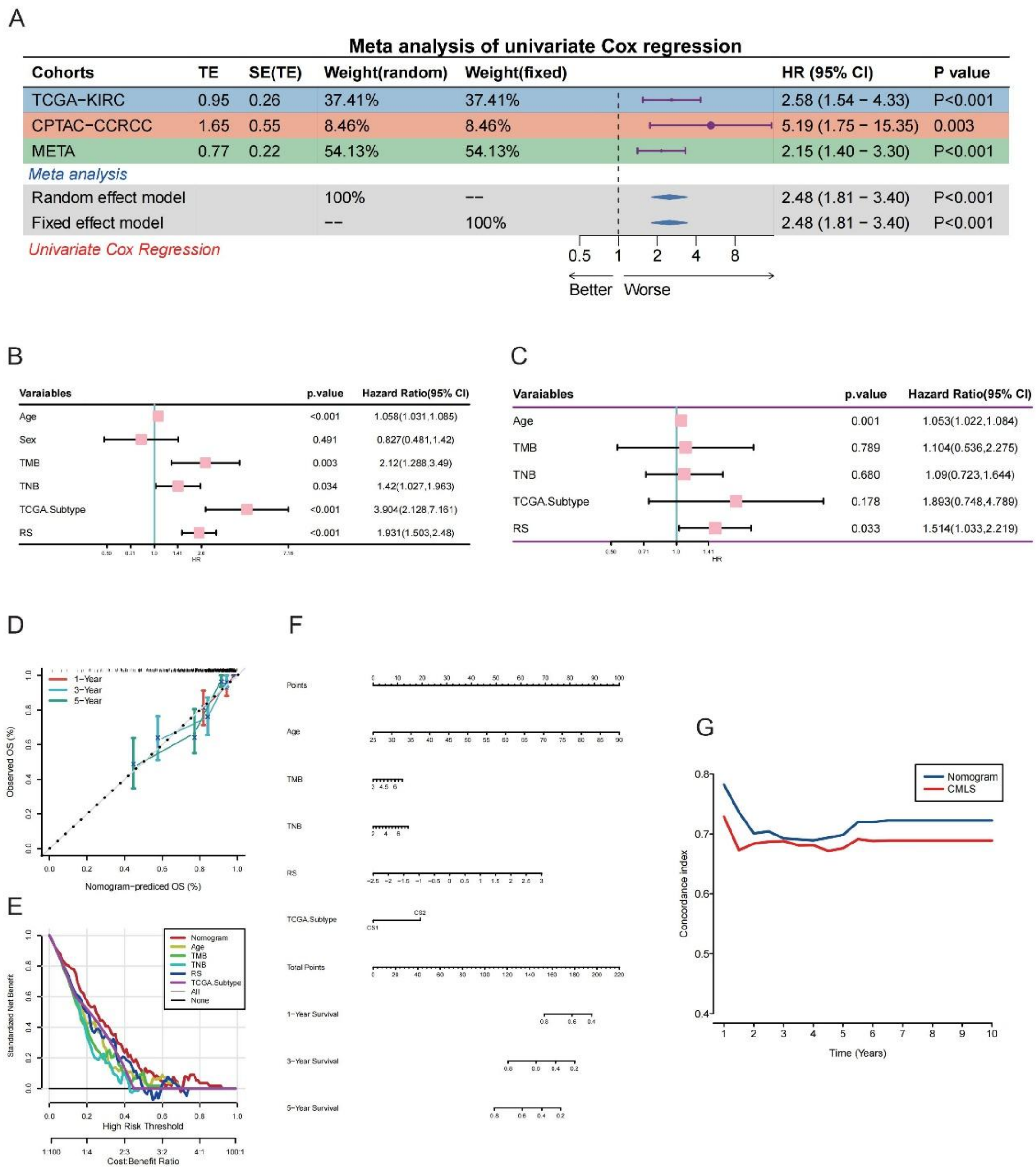

Figure S2. Clinical translation of the CMLS and construction of a composite prognostic nomogram.

(A) Integrated meta-analysis of univariate Cox regression models verifying the pan-cohort predictive capacity of the CMLS across the TCGA-KIRC, Meta-ccRCC, and CPTAC-ccRCC populations.

(B) Univariate assessment correlating the prognostic signature alongside traditional clinicopathological variables.

(C) Rigorous multivariate Cox analysis confirming the CMLS as a robust, independent determinant of survival.

(D) Calibration plots assessing the alignment between nomogram-estimated survival probabilities and actual clinical observations.

(E) Decision Curve Analysis (DCA) quantifying the net clinical utility of the composite model across a continuum of risk thresholds.
(F) A quantitative clinical nomogram designed to accurately forecast 1-, 3-, and 5-year overall survival outcomes.
(G) Time-dependent C-index tracking the sustained discriminative superiority of the multivariate predictive framework.

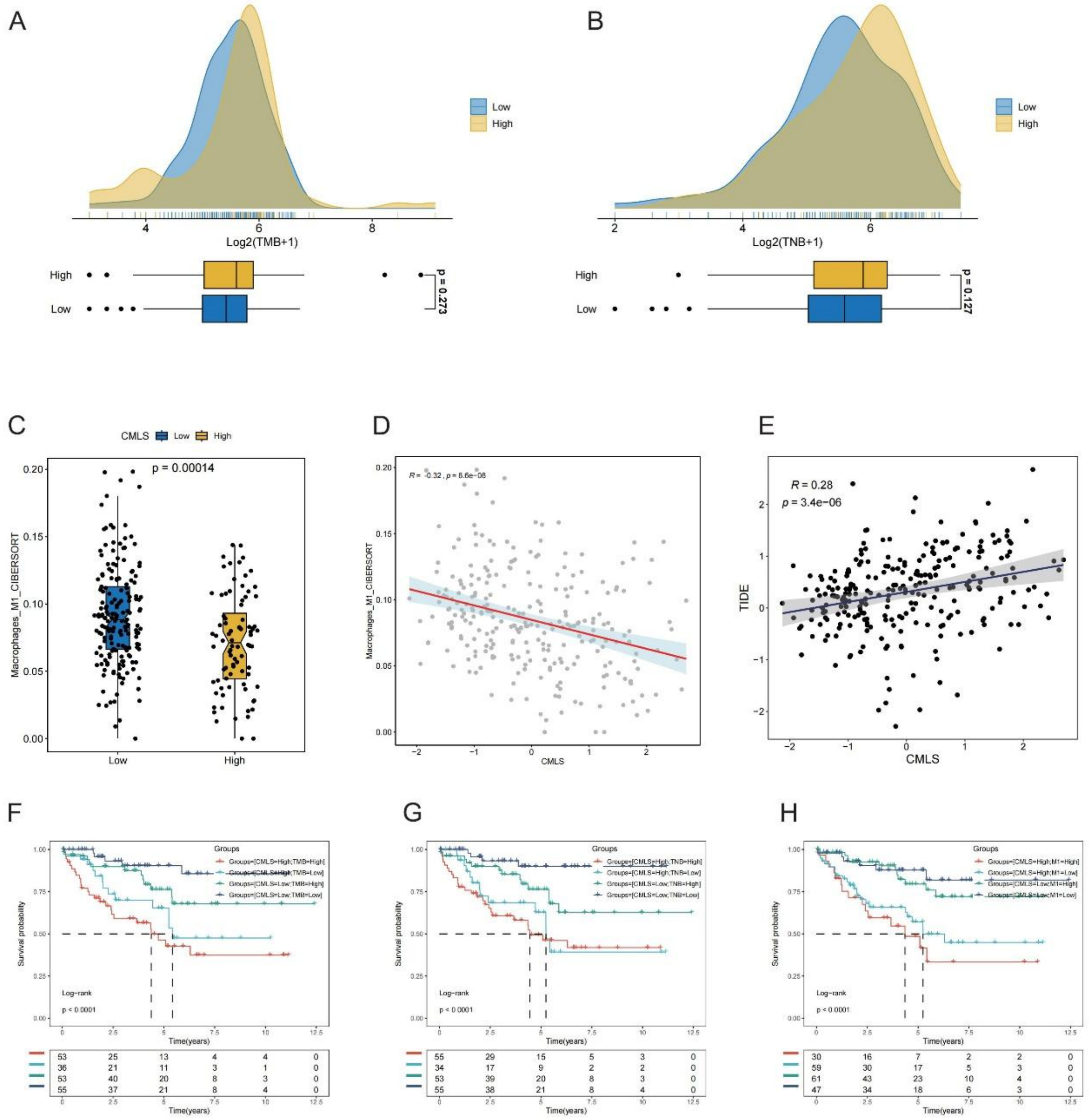

Figure S3. Crosstalk between the CMLS risk strata, conventional immunogenic biomarkers, and the microenvironment.

(A) Comparative distribution of macroscopic tumor mutational burden (TMB) across the distinct CMLS subsets.

(B) Evaluation of tumor neoantigen burden (TNB) disparities between the high- and low-CMLS classifications.

(C) Differential infiltration patterns of anti-tumorigenic M1 macrophages dictated by CMLS status.

(D) Correlative analysis detailing the specific association linking CMLS scores to M1 macrophage abundance.

(E) Linear modeling demonstrating the relationship between the prognostic signature and overall TIDE predictive scores.

(F–H) Combinatorial Kaplan-Meier survival evaluations integrating the CMLS alongside traditional TMB (F), TNB (G), and M1 macrophage (H) status to refine prognostic stratification.
